# Ly6C^hi^ Monocytes and Their Macrophage Descendants Regulate Neutrophil Function and Clearance in Acetaminophen-Induced Liver Injury

**DOI:** 10.3389/fimmu.2017.00626

**Published:** 2017-06-01

**Authors:** Nadine Graubardt, Milena Vugman, Odelia Mouhadeb, Gabriele Caliari, Metsada Pasmanik-Chor, Debby Reuveni, Ehud Zigmond, Eli Brazowski, Eyal David, Lousie Chappell-Maor, Steffen Jung, Chen Varol

**Affiliations:** ^1^The Research Center for Digestive Tract and Liver Diseases, Tel-Aviv Sourasky Medical Center and Sackler School of Medicine, Tel-Aviv University, Tel Aviv, Israel; ^2^Department of Immunology, Weizmann Institute of Science, Rehovot, Israel; ^3^Department of Clinical Microbiology and Immunology, The Sackler School of Medicine, Tel-Aviv University, Tel Aviv, Israel; ^4^Bioinformatics Unit, G. S. Wise Faculty of Life Science, Tel-Aviv University, Tel Aviv, Israel

**Keywords:** macrophages, monocytes, neutrophils, drug-induced liver injury, liver immunology

## Abstract

Monocyte-derived macrophages (MoMF) play a pivotal role in the resolution of acetaminophen-induced liver injury (AILI). Timely termination of neutrophil activity and their clearance are essential for liver regeneration following injury. Here, we show that infiltrating Ly6C^hi^ monocytes, their macrophage descendants, and neutrophils spatially and temporally overlap in the centrilobular necrotic areas during the necroinflammatory and resolution phases of AILI. At the necroinflammatory phase, inducible ablation of circulating Ly6C^hi^ monocytes resulted in reduced numbers and fractions of reactive oxygen species (ROS)-producing neutrophils. In alignment with this, neutrophils sorted from monocyte-deficient livers exhibited reduced expression of NADPH oxidase 2. Moreover, human CD14^+^ monocytes stimulated with lipopolysaccharide or hepatocyte apoptotic bodies directly induced ROS production by cocultured neutrophils. RNA-seq-based transcriptome profiling of neutrophils from Ly6C^hi^ monocyte-deficient versus normal livers revealed 449 genes that were differentially expressed with at least twofold change (*p* ≤ 0.05). In the absence of Ly6C^hi^ monocytes, neutrophils displayed gene expression alterations associated with decreased innate immune activity and increased cell survival. At the early resolution phase, Ly6C^hi^ monocytes differentiated into ephemeral Ly6C^lo^ MoMF and their absence resulted in significant accumulation of late apoptotic neutrophils. Further gene expression analysis revealed the induced expression of a specific repertoire of bridging molecules and receptors involved with apoptotic cell clearance during the transition from Ly6C^hi^ monocytes to MoMF. Collectively, our findings establish a phase-dependent task division between liver-infiltrating Ly6C^hi^ monocytes and their MoMF descendants with the former regulating innate immune functions and cell survival of neutrophils and the later neutrophil clearance.

## Introduction

One of the most peculiar characteristics of the liver is the regenerative process that occurs in response to damage and/or injury. Key players of this healing reaction are recruited monocytes and macrophages that undergo marked phenotypic and functional changes that, which license them to promote the initiation, maintenance, and resolution phases of tissue repair (1). Macrophages are an integral functional component of the liver during homeostasis (2), however, their contribution to liver inflammation and resolution remains under debate, with a plethora of studies reporting on both deleterious and hepatoprotective functions of these cells (3–11). The controversy likely arises from the heterogeneity of the liver macrophage compartment, comprising both liver resident Kupffer cells (KCs) and monocytic infiltrates with considerable functional plasticity. Specifically, proinflammatory activity has been attributed to liver infiltrating Ly6C^hi^ monocytes in various acute and chronic liver injury models (12–16). In a model of reversible hepatic fibrosis, these monocytes advance fibrogenesis (7), yet at the resolution phase, the same cells give rise to distinct Ly6C^lo^ prorestorative macrophages that actively promote liver regeneration (5, 8). Similar functional dichotomy was reported in the healing of other tissue-specific injuries such as heart (17), skeletal muscle (18), spinal cord (19), retina (20), and sterile wounds (21).

Others and we have recently embarked on the phenotypic, ontogenic, and molecular definition of the liver macrophage compartment following acute injury caused by overdose of acetaminophen [*N*-acetyl-*p*-aminophenol (APAP); paracetamol] (22, 23). KCs are significantly reduced upon APAP-induced liver injury (AILI) and recover by self-renewal at the resolution phase (22, 23). In contrast, Ly6C^hi^ monocytes are recruited in a CCR2- and M-CSF-mediated manner to become the predominant macrophage subset at the necroinflammatory phase (24 h postchallenge) and subsequently differentiate into ephemeral Ly6C^lo^ monocyte-derived macrophages (MoMF) at the early resolution phase (starting from 48 h) (22). The conditional selective ablation of Ly6C^hi^ monocytes and consequently of their MoMF descendants results in impaired recovery from injury suggesting their pivotal involvement in the resolution from liver damage (22). These results extended earlier studies showing impaired liver resolution following AILI in *Ccr2*^−^*^/^*^−^ mice in which liver monocyte recruitment was diminished (6, 24).

Extensive cell necrosis during AILI initiates an innate inflammatory response with neutrophil recruitment (25). Neutrophils facilitate the recovery from tissue injury by production of lytic enzymes and reactive oxygen species (ROS) necessary for the removal of damaged tissue and necrotic cells (26–29). However, impaired regulation of this neutrophil activity leading to excessive ROS production can cause collateral liver damage. Indeed, neutrophils can aggravate tissue damage in various liver injury models, including hepatic ischemia–reperfusion injury (30), endotoxemia (31, 32), alcoholic hepatitis (33), and bile duct ligation (34), though their role in the pathogenesis of AILI has remained controversial (35–38). Therefore, timely termination of neutrophil activity and their clearance are essential for the resolution of liver injury.

Previous studies suggested that phagocytes including neutrophils, monocytes, and macrophages cooperate during the onset, progression, and resolution of inflammation (28). Yet, the specific interplay of these cells during acute liver injury has remained elusive. Here, we demonstrate that liver infiltrating monocytes, MoMF, and neutrophils spatially and temporally overlap in the centrilobular necrotic areas following AILI. Moreover, we show that Ly6C^hi^ monocytes directly promote ROS production by neutrophils localizing in their proximity. RNA-seq transcriptomic profiling of neutrophils extracted at 24 h following AILI from normal versus Ly6C^hi^ monocyte-deficient livers suggests that monocytes activate neutrophils innate immune pathways and facilitate their apoptosis. At the resolution phase, monocytes differentiate into MoMF and promote neutrophil clearance.

## Materials and Methods

### Mice

The following 8- to 12-week-old male mouse strains were used: C57BL/6J wild-type mice were purchased from Harlan Laboratories (Rehovot, Israel) and *Cx3cr1^gfp/+^*mice (39) were bred at the Sourasky Medical Center animal facility and originally provided by Prof. Steffen Jung, the Weizmann Institute of Science. If not noted otherwise, mice had free access to standard mouse food.

### Acetaminophen-Induced Liver Injury (AILI)

Mice were fasted overnight for 12 h prior to intraperitoneal (i.p.) administration of 300 mg/kg Acetaminophen (APAP, Sigma-Aldrich, USA). Water was returned concomitantly with APAP administration and the food at 2 h later.

### Quantification of Hepatic Damage

Liver samples were obtained at 24 h after AILI, fixed (4% paraformaldehyde), paraffin-embedded, sectioned, and stained with H&E. Pathologic evaluation was performed by a pathologist (Eli Brazowski). Necrosis was scored as 0 (no necrosis), 1 (spotty necrosis), 2 (confluent, zone 3 necrosis), 3 (confluent, zone 2 plus 3 necrosis), or 4 (panlobular necrosis). Bridging necrosis was scored as 0 (absent) or 1 (present) and ballooning of hepatocytes as 0 (absent), 1 (mild), 2 (moderate), or 3 (severe). Serum alanine aminotransferase (ALT) and aspartate aminotransferase (AST) levels were measured using a Hitachi 747 Automatic Analyzer.

### MC-21-Mediated Ablation of MoMF

When monocyte ablation was required, mice received an i.p. injection of 400 µL anti-mouse CCR2 mAb (clone MC-21)-conditioned media (29 µg Ab/mL). The injections were performed, starting at 12 h prior to APAP challenge and every 24 h, till the time of sacrifice.

### Isolation of Hepatic Non-Parenchymal Cells

Isolation of hepatic non-parenchymal cells was performed as previously described (22). In brief, mice were anesthetized and perfused livers were collected, cut into small fragments, and incubated with 5 mL digestion buffer composed by 5% fetal bovine serum (Biological Industries, Israel), 0.5 mg/mL Collagenase VIII from *Clostridium histolyticum* (Sigma-Aldrich, USA), 0.1 mg/mL Deoxyribonuclease I from bovine pancreas (Sigma-Aldrich, USA) in Dulbecco’s phosphate-buffered saline with calcium and magnesium (PBS^+/+^, Biological Industries, Israel), in a shaker-incubator at 250 rpm, 37°C for 45 min. The samples were then subjected to three cycles of washing with Dulbecco’s phosphate-buffered saline without calcium and magnesium (PBS^−/−^) at 400 rpm, 4°C for 5 min from which the supernatant was kept, omitting the parenchymal cell pellet. Subsequently, the supernatant was centrifuged at 1,400 rpm, 4°C for 5 min and the cell pellet was lysed for erythrocytes by 2 min incubation with ACK buffer composed by 0.15 M NH_4_Cl, and 0.01 M KHCO_3_, and washed with PBS^−/−^.

### Flow Cytometry Analysis

The following antibodies were used for flow cytometry analysis (dilutions are indicated): anti-mouse CD45 (clone 30-F11, 1:100), anti-mouse/human CD11b (clone M1/70, 1:300), anti-mouse Ly6C (clone HK1.4, 1:300), anti-mouse MHCII (clone M5/114.15.2, 1:200), anti-mouse CD64 (clone X54-5/7.1, 1:50), anti-mouse Ly6G (clone 1A8, 1:100), and anti-mouse Tim4 (clone RMT4-54, 1:50), which were purchased from BioLegend, San Diego, CA, USA. Anti-mouse F4/80 (clone A3-1, 1:50) was purchased from BIORAD. The staining for ROS was performed with 0.1 mM of 2,7-dichlorodihydrofluoresceindiacetate (Molecular Probes Invitrogen). Staining for apoptosis and necrosis markers with Annexin V and propidium iodide was performed with MEBCYTO-Apoptosis Kit (MBL International Corporation). Cells were analyzed with BD FACSCanto™ II (BD Bioscience). Flow cytometry analysis was performed using FlowJo software (TreeStar, Ashland, OR, USA).

### Immunohistochemistry and Immunofluorescence

Ly6G-hematoxylin immunostaining was performed on paraffin-embedded liver sections. For antigen retrieval, slides were placed in 10 mM citric buffer at pH 6 in autoclave at 100 kP. Next, the incubation slides were transferred to H_2_O_2_ and DDW and then processed with Optimax Wash Buffer (BioGenex, USA). Sections were stained with primary antibody anti-mouse Ly6G (clone 1A8, BioLegend, 1:100) in CAS-Block (Invitrogen, USA) for 24 h at 4°C in a wet chamber. After incubation, sections were washed in Optimax Wash Buffer and treated with MACH 3 Mouse Probe and MACH 3 Mouse HRP-Polymer (BIOCARE MEDICAL), according to the manufacturer’s protocol. Peroxidase substrate kit, 3,3-diaminobenzidine tetrahydrochloride (Vector Laboratories) was added to the sections in order to develop the color. Cx3cr1-GFP and Ly6G immunofluorescent staining was performed on frozen liver sections of 13 µm. *Z*-stacking, imaging was performed on 20 µm thick slides. Slides were incubated in cold acetone for 6 min and dried at room temperature. Following washing, slide sections were blocked with Normal Donkey Serum (Jackson ImmunoResearch, Inc.) for 2 h at room temperature. Samples were stained with primary anti-Ly6G-A647 antibody (clone 1A8, 1:100, BioLegend, San Diego, CA, USA) and for 24 h at 4°C followed by double washing. Subsequently, slides were washed with PBST and mounted with Fluorescent Mounting Medium with or without 4,6-diamidino-2-phenylindole (GBI Labs). Images were taken with ZEISS Confocal Microscope (MicroImaging GmbH, ZEISS, Germany). Processing was performed with ZEN 2010 software.

### Quantitative Real-Time PCR

CD45^+^CD11b^+^Ly6G^hi^Ly6C^−/lo^CX_3_CR1^−^ neutrophils were sorted from livers of mice treated with PBS or MC-21, at 24 h following AILI. RNA was isolated using the Ambion Dynabeads^®^ mRNA DIRECT™ Kit, catalog number 61012. Fifty thousand neutrophils were sorted directly into the Lysis/Binding buffer supplied with the kit and isolation was performed according to the manufacturer’s instructions. RNA was then reverse transcribed with the AffinityScript cDNA synthesis kit (Agilent Technologies). PCRs were performed with the SYBER green PCR Master Mix (Applied Biosystems) and the Applied Biosystems 7300 Real-Time PCR machine. The *Cybb* gene expression was compared with ribosomal protein, large PO (*Rplp0*) housekeeping gene. Primer sequences (forward and reverse, respectively) were: RPLP0, 5′-T CCAGCAGGTGTTTGACAAC-3′ and 5′-CCATCTGCAGACACACACT-3′; CYBB, 5′-CCTCTACCAAAACCATTCGGAG-3′ and 5′-CTGTCCACGTACAATTC GTTCA-3′.

### Human Cell Purification and Culture

CD14^+^ monocytes were isolated (>90% purity) from peripheral blood of healthy donors by negative selection using the Monocyte Isolation Kit II Human (Miltenyi Biotec, Germany). The enriched monocyte fraction was suspended for 2 h in RPMI 1640 medium, supplemented with Penicillin/Streptomycin and l-Glutamine only, or activated for 2 h with either 100 ng/mL of *Escherichia coli* lipopolysaccharide (LPS, Sigma-Aldrich, USA) or human hepatocyte apoptotic bodies (generated by exposure of Hep G2 human hepatocellular carcinoma cell line to UV light 0–100 mJ/cm^2^, 142 s). Monocytes were then double washed to exclude direct activation of neutrophils by LPS or apoptotic hepatocytes. In case of LPS stimulation, 50 µg/mL of Polymyxin B (PMB sulfate salt, Sigma-Aldrich, USA) were added to the neutrophil cultures in order to ensure neutralization of LPS residuals, which may directly affect neutrophil activity. Neutrophils were purified (>90% purity) from peripheral blood of healthy donors by Ficoll gradient (Ficoll-Paque™ PLUS, GE Healthcare) as previously described (40). The purified neutrophils were then cultured in 96-well tissue culture round bottom plates at 37°C with the stimulated monocyte cells or with fresh cell-free supernatants extracted from these monocyte cultures. In both cases, non-stimulated monocytes and sole neutrophil cultures were used as controls. After 2 h incubation, pelleted cells were stained with anti-human neutrophil marker CD66b antibody (clone G10F5, BD Biosciences) and for ROS production by flow cytometry. Healthy donors were enrolled after providing informed consent in accordance with the ethical standards on Human Experimentation and the Declaration of Helsinki (#920080132).

### RNA-seq

For RNA-seq 50,000 neutrophil cells per liver were sorted by FACSAria directly into a 1.7 mL microtube containing 50 µL lysis buffer [RNase-free H_2_O, 0.2% Triton-X (Roth) and 0.4 U/μL RNasin (Promega)]. Next, the tube was centrifuged, snap frozen on dry ice and stored at −80°C. RNA-seq library generation, sample preparation and analysis were carried out as previously described (41).

### RNA-seq Processing and Analysis

Four control neutrophil samples and three neutrophil samples extracted from livers of MC-21-treated mice were analyzed by NGS (Illumina NextSeq 500). FastQ files were indexed and mapped for Mm9 genome assembly using HISAT 0.1.5 (42). SAM files were converted to BAM using SAMtools (43). BAM files were analyzed using Partek Genomics Suite 6.6 software.^1^ Gene RPKM (Reads Per Kilobase of transcript per Million mapped reads) (44) normalized reads were obtained, and differentially expressed genes were filtered with cutoffs of *p* < 0.05 (unpaired, two-tailed *t*-test) and fold-change difference of at least twofold. Functional enrichment analyses were performed using DAVID tool (45). All RNA-seq data have been deposited at the National Center for Biotechnology Information Gene Expression Omnibus public database under accession no. GSE95182.

### Gene-Expression Data Mining

Gene expression of apoptotic cell bridge molecules and receptors was extracted out of our previously published database (22) deposited at the National Center for Biotechnology Information Gene Expression Omnibus public database^2^ under accession number GSE55606. Heat maps were performed using Partek Genomics Suite software.

### Statistical Analysis

Data were analyzed by unpaired, two-tailed *t*-test with GraphPad Prism 5.0b (San Diego, CA, USA). Data are presented as mean ± SEM; values of *p* < 0.05 were considered statistically significant.

### Ethics Statement

Studies with human cells were carried out in accordance with the recommendations of Tel-Aviv Sourasky Medical Center Helsinki committee. All subjects gave written informed consent in accordance with the Declaration of Helsinki. The protocol was approved by the Tel-Aviv Sourasky Medical Center Helsinki committee (protocol # 920080132). All mouse studies were carried out in accordance with the recommendations of Tel-Aviv Sourasky Medical Center ethical committee for animal studies. The protocols were approved by the local committee (protocol # 8-3-13 and 29-10-15).

## Results

### Liver Infiltrating Monocytes, MoMF, and Neutrophils Display Overlapping Migratory Behavior following AILI

**Figure 1:**
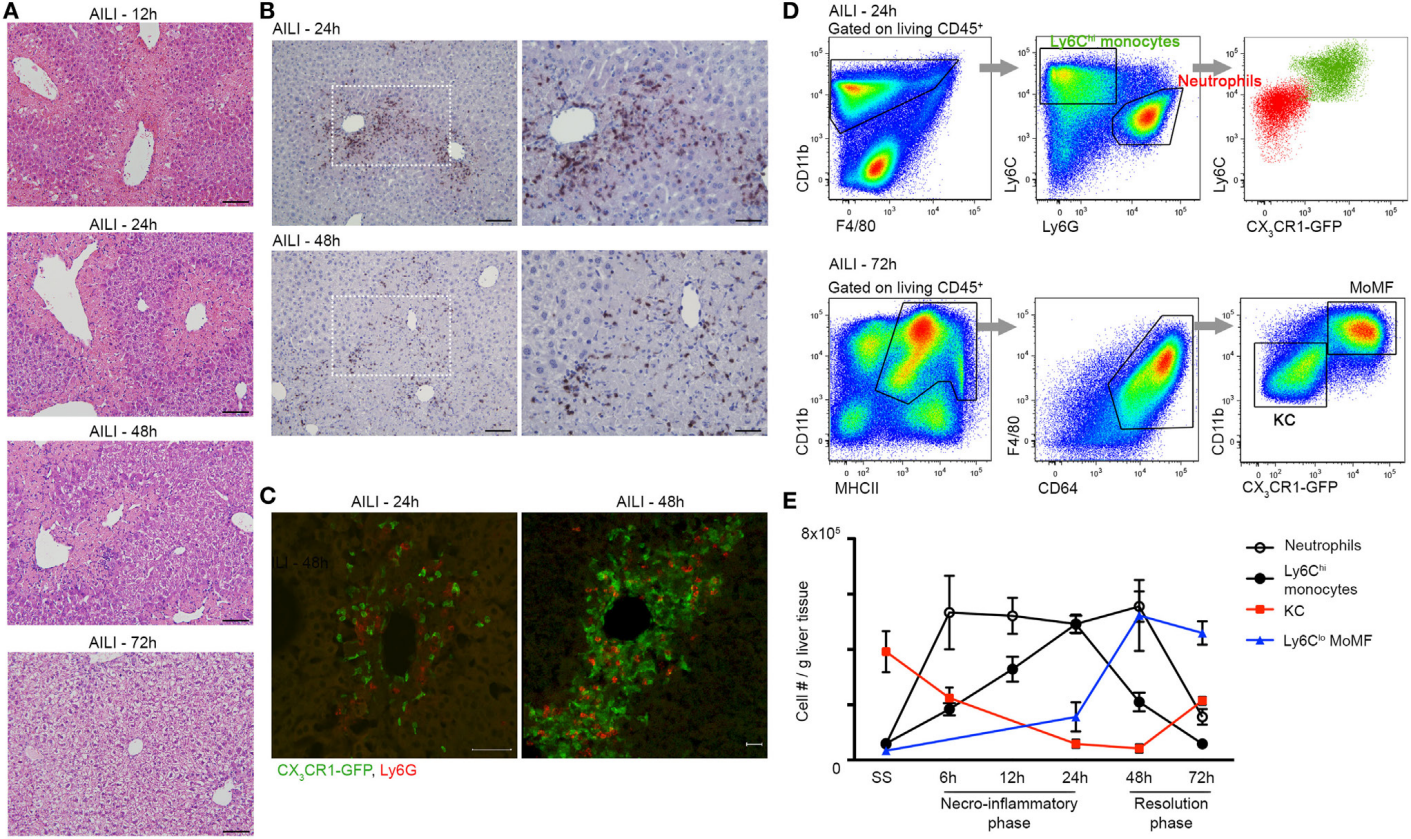
**Liver infiltrating Ly6C^hi^ monocytes, monocyte-derived macrophage (MoMF), and neutrophils spatially and temporally coincide at the inflammatory and resolution phases of acetaminophen-induced liver injury (AILI)**. **(A)** Hematoxylin and eosin (H&E) staining of liver sections at 12, 24, 48, and 72 h following AILI. Original magnification ×20. Bars, 100 µm. **(B)** Immunostaining for Ly6G and hematoxylin on paraffin-embedded liver sections at 24 and 48 h after AILI. Original magnifications ×20 (left image) and ×40 (right image). Bars, 100 µm (×20) and 85 µm (×40). **(C)** Immunofluorescence staining at 24 and 48 h after AILI in *Cx3cr1^gfp/+^* mice. At 48 h, 3D-reconstruction was performed of Z-stacks acquired from 20 µm thick slides. In green are infiltrating monocyte-derived cells and in red are neutrophils, which are stained for Ly6G. Original magnification ×20. Bars, 50 µm for 24 h and 20 µm for 48 h. **(D)** Flow cytometry-based definition of non-parenchymal *Cx3cr1^gfp/+^* liver cells isolated at 24 h (Ly6C^hi^ monocytes and neutrophils) or 72 h [MoMF and Kupffer cell (KC)] following AILI. **(E)** Flow cytometry-based analysis of the dynamics of neutrophils and liver-macrophage subsets at steady state (SS) and at different time points following AILI: Ly6G^+^ neutrophils (white), infiltrating Ly6C^hi^CX_3_CR1-GFP^+^ monocytes (black), resident CX_3_CR1-GFP^neg/lo^ KC (red), and Ly6C^lo^CX_3_CR1-GFP^hi^ MoMF (blue), presented as cell counts normalized per liver tissue mass in gram. Data are presented as mean ± SEM; *n* ≥ 5 mice for each time point. Experiments were repeated at least three times.

Acetaminophen-Induced Liver Injury is associated with massive liver infiltration of monocytes and neutrophils (6, 22, 35, 37). To dissect the kinetics of these phagocyte infiltrates and probe for potential communication between them, we performed a detailed histological analysis of liver sections of APAP-challenged C57BL/6 mice. Hematoxylin and eosin (H&E) staining discovered extensive hepatocyte damage, with bridging necrosis, ballooning degeneration, and massive immune cell infiltration at 12, 24, and 48 h following APAP administration. At 72 h, liver regeneration was already initiated, though hepatocyte ballooning was still evident (Figure 1A). Ly6G-Hematoxylin immunostaining revealed that neutrophils infiltrate the centrilobular necrotic areas at 24 h, and are still apparent at early resolution phase at 48 h (Figure 1B). To visualize the mononuclear infiltrates, we took advantage of *Cx3cr1^gfp/+^* reporter mice (39), in whose livers monocyte-derived cells, but not resident KC, are GFP-labeled (22, 46). Immunofluorescent staining of *Cx3cr1^gfp/+^* liver sections revealed that Ly6G^+^ neutrophils and CX_3_CR1-GFP^+^ monocyte-derived cells colocalize within the centrilobular necrotic areas at 24 and 48 h following AILI (Figure 1C, Movie S1 in Supplementary Material).

To accurately follow the migration kinetics of neutrophils, monocytes, and macrophage subsets in the injured *Cx3cr1^gfp/+^* livers at various time points following AILI, we performed multiparameter flow cytometry analysis on purified non-parenchymal liver cells (22). Neutrophils were defined as CD11b^+^Ly6C^lo^CX_3_CR1-GFP^−^Ly6G^+^ cells, while monocytes were defined as CD11b^+^Ly6C^hi^CX_3_CR1-GFP^+^MHCII^−^Ly6G^−^ cells. KC and MoMF expressed similar levels of the macrophage markers F4/80, CD64 (FcγR), and MHCII, but could clearly be discriminated according to presence and absence of the CX_3_CR1-GFP label (Figure 1D). Neutrophils and monocytes displayed similar recruitment kinetics and accumulated to be the dominant phagocyte populations in the necroinflammatory phase (24 h). Neutrophils are the first cell type to infiltrate tissue after injury. Indeed, neutrophil infiltration preceded that of monocytes at 6 and 12 h after AILI (Figure 1E). At early resolution phase (48 h), neutrophils were still dominant, while many of the Ly6C^hi^ monocytes have already differentiated toward Ly6C^lo^ MoMF. At the resolution phase (72 h), neutrophils were scarcely present and MoMF turned to be the major macrophage subset. Resident KCs were significantly reduced at the necroinflammatory phase of AILI and started to repopulate at the resolution phase (Figure 1E). Collectively, these results show considerable overlap in the infiltration patterns of liver neutrophils and monocytes with respect to location and time.

### Liver Infiltrating Ly6C^hi^ Monocytes Induce ROS Production by Neutrophils

**Figure 2:**
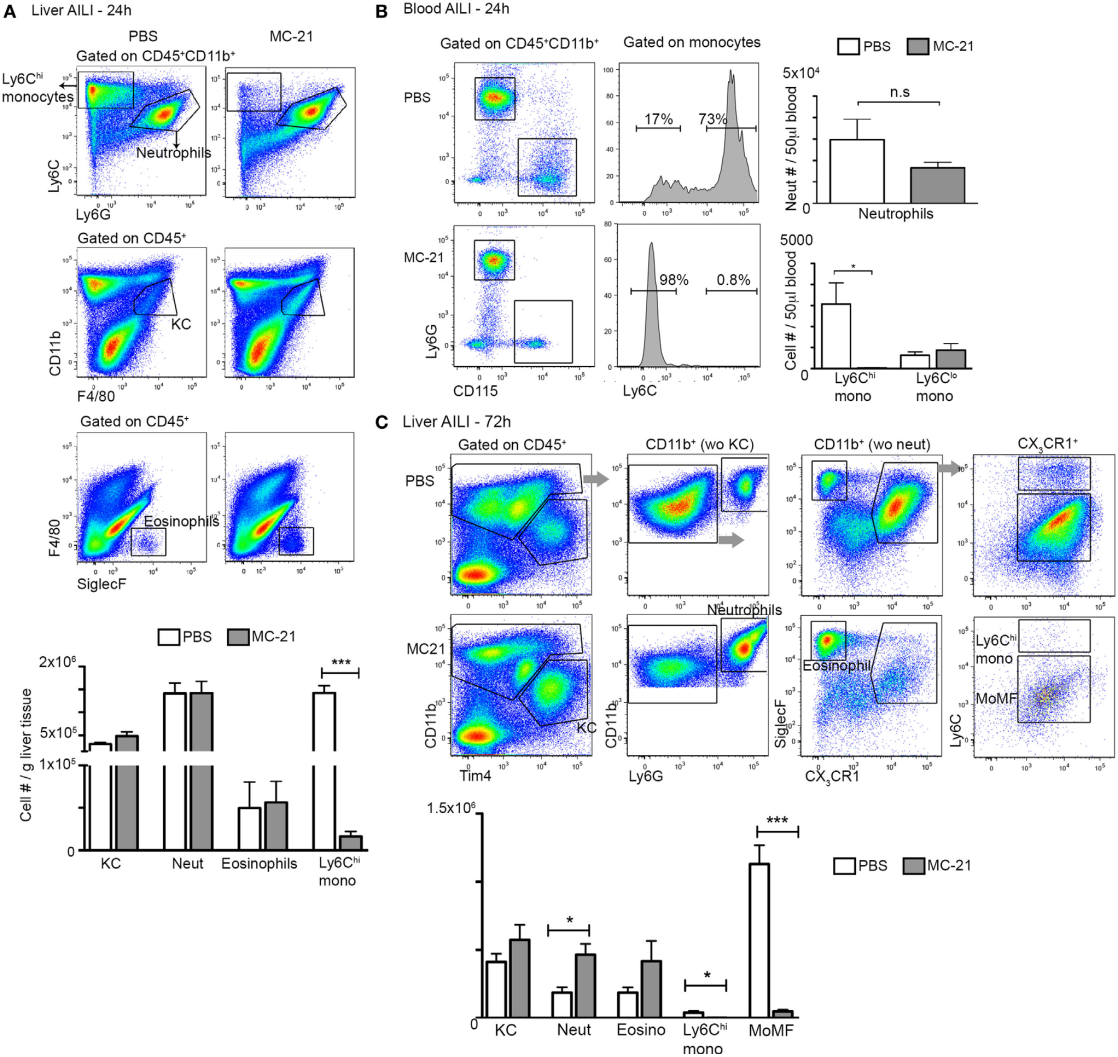
**MC-21 treatment induces the specific ablation of Ly6C^hi^ monocytes and MoMF**. **(A)** Flow cytometry analysis of hepatic non-parenchymal cells at 24 h following acetaminophen-induced liver injury (AILI) in mice injected with MC-21 or phosphate-buffered saline (PBS). Below, graphical summary of Kupffer cell (KC), neutrophil, eosinophil, and Ly6C^hi^ monocyte cell numbers normalized for liver tissue mass. Note the specific depletion of Ly6C^hi^ monocytes. **(B)** Flow cytometry analysis of peripheral blood of control versus MC-21-treated mice showing Ly6C^hi^ and Ly6C^lo^ CD115^+^ monocyte subsets as well as CD115^−^Ly6G^hi^ neutrophils. Next, graphical summaries showing the cell numbers per 50 µL blood of neutrophils and Ly6C^hi^ and Ly6C^lo^ monocyte subsets at 24 h following AILI. **(C)** Flow cytometry analysis of hepatic non-parenchymal cells at 72 h following AILI in mice injected with MC-21 or PBS. Below, graphical summary of KC, neutrophil, eosinophil, Ly6C^hi^ monocyte and monocyte-derived macrophage (MoMF) cell numbers normalized for liver tissue mass. Note the specific depletion of Ly6C^hi^ monocytes and their MoMF descendants. Data are presented as mean ± SEM; *n* ≥ 5 mice for each time point. Data were analyzed by unpaired, two-tailed *t*-test, comparing each time between PBS and MC-21-treatments, and are presented as mean ± SEM with significance: *p < 0.05 and ***p < 0.001 (*n* ≥ 5 mice/group). Experiments were repeated at least twice.

The spatial and temporal colocalization of liver infiltrating neutrophils and Ly6C^hi^ monocytes following AILI prompted us to determine whether these cells are functionally intertwined. Ly6C^hi^ monocyte egress out of the bone marrow to the circulation is CCR2-dependent (47), and are hence amenable to conditional *in vivo* ablation already at the circulation by the anti-CCR2 antibody MC-21 (19, 48, 49). Efficient and specific ablation of circulating and liver infiltrating Ly6C^hi^ monocytes and of their MoMF descendants by MC-21 was confirmed by flow cytometry at 24 and 72 h following AILI, respectively (Figures 2A–C). At 24 h following AILI, MC-21-induced monocyte ablation had no effect on the numbers of liver resident KC, infiltrating neutrophils, and eosinophils (Figure 2A), as well as on the abundance of circulating neutrophils and Ly6C^lo^ monocytes (Figure 2B). At 72 h post AILI, MC-21-mediated ablation had no affect on Tim4^+^ KC repopulation and eosinophil recruitment, but there was a significant increase in neutrophil number (Figure 2C). We have previously reported that the inducible ablation of Ly6C^hi^ monocytes and their MoMF descendants impairs liver resolution at 48 h following AILI (22). Corroborating these results, blinded histopathological assessment of livers extracted from MC-21 treated mice revealed extended necrotic damage specifically at 48 h following AILI (Figure S1A in Supplementary Material) with significant increase in the pathological score (Figure S1B in Supplementary Material). Importantly, we could not detect any significant impact on hepatic damage at the necroinflammatory phase as manifested by similar histopathological score at 12 and 24 h post-AILI (Figures S1A,B in Supplementary Material) and similar levels of the liver enzymes ALT/AST in the serum (Figures S1C,D in Supplementary Material) at 24 h post-AILI. Liver enzyme levels were profoundly and gradually reduced starting at 48 h post AILI with no significant impact for MC-21-mediated ablation (Figures S1C,D in Supplementary Material). Therefore, these results point to MC-21-induced Ly6C^hi^ monocyte ablation as being a suitable model for studying monocyte effects on neutrophil activity and clearance during AILI.

**Figure 3:**
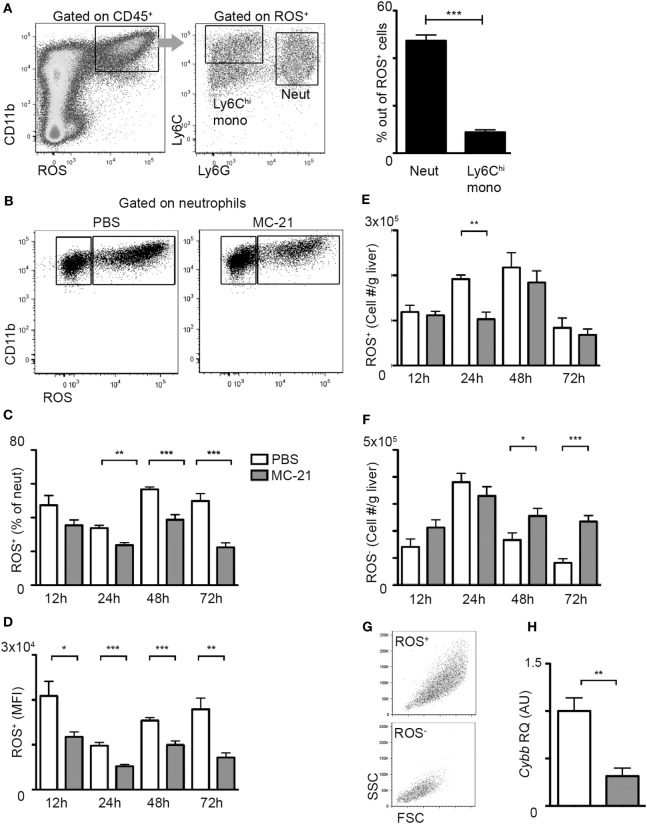
**Reduced neutrophil-derived reactive oxygen species (ROS) production in the absence of Ly6C^hi^ monocytes**. **(A)** Flow cytometry and graphical representation demonstrating the definition of CD11b^+^ ROS^+^ cells at acetaminophen-induced liver injury (AILI)-24 h livers and the fraction of Ly6C^hi^ monocytes and Ly6G^+^ neutrophils out of these cells. **(B)** Flow cytometry analysis at AILI-24 h livers showing ROS staining in neutrophils gated as CD45^+^CD11b^+^Ly6G^+^Ly6C^lo^ cells. **(C–F)** Graphical summaries of flow cytometry analyses of injured livers of phosphate-buffered saline (PBS) (white) versus MC-21-treated (gray) mice at 12, 24, 48, and 72 h following AILI showing the **(C)** fraction of ROS^+^ neutrophils out of total living neutrophils, **(D)** mean fluorescence intensity (MFI) of ROS out of ROS^+^ neutrophil population, and **(E)** the numbers of ROS^+^ and **(F)** of ROS^−^ neutrophils normalized per liver tissue mass in gram. **(G)** Flow cytometry representation of size (FSC) and granularity (SSC) parameters of ROS^+^ and ROS^−^ neutrophil populations. Note that ROS^−^ cells are smaller and less granular. **(H)** Graphical representation of quantitative RT-PCR gene expression analysis of *Cybb*, comparing neutrophils from PBS (white) and MC-21-treated (gray) livers. For A and C-F, data were analyzed by unpaired, two-tailed *t*-test, comparing each time livers from PBS versus MC-21-treated mice and are presented as mean ± SEM with significance: *p < 0.05, **p < 0.01, ***p < 0.001 (*n* ≥ 5 mice/group for each time point). For H, data were analyzed by unpaired, two-tailed t-test, comparing sorted neutrophils from livers of PBS (white) versus MC-21-treated (gray) mice and are presented as mean ± SEM with significance: **p < 0.01 (*n* ≥ 4 mice/group).

A hallmark of neutrophil recruitment to sites of injury is their synthesis of ROS, which can cause collateral tissue damage, if not restrictively controlled (26–29). Flow cytometry analysis at the necroinflammatory phase uncovered that ROS production is restricted to CD11b^+^ myeloid cells; among them the fraction of Ly6G^+^ neutrophils was profoundly greater than that of Ly6C^hi^ monocytes (Figure 3A). Interestingly, monocyte ablation resulted in a significantly reduced percentage of ROS^+^ neutrophils during both the necroinflammatory and the resolution phase after injury (Figures 3B,C). Moreover, ROS mean fluorescence intensity out of ROS^+^ neutrophils was reduced at all analyzed time points (Figure 3D). With respect to the numbers of ROS^+^ neutrophils, there was a significant decrease in response to Ly6C^hi^ monocyte-ablation specifically at the 24 h time point of the necroinflammatory phase (Figure 3E). In contrast, we observed a significant accumulation of ROS^−^ cells at the resolution phase (48 and 72 h) (Figure 3F), which were smaller and less granular than ROS^+^ cells (Figure 3G). In alignment with the reduced ROS production in neutrophils at the necroinflammatory phase, liver neutrophils sorted at 24 h following AILI displayed marked reduction in the gene encoding for NADPH Oxidase 2 (*Cybb*) (Figure 3H), a key driver of ROS production in neutrophils (50). Collectively, these data suggest that liver infiltrating Ly6C^hi^ monocytes promote ROS-production by neutrophils at the necroinflammatory phase of AILI.

### CD14^+^ Human Monocytes Directly Activate ROS Production in Neutrophils

**Figure 4:**
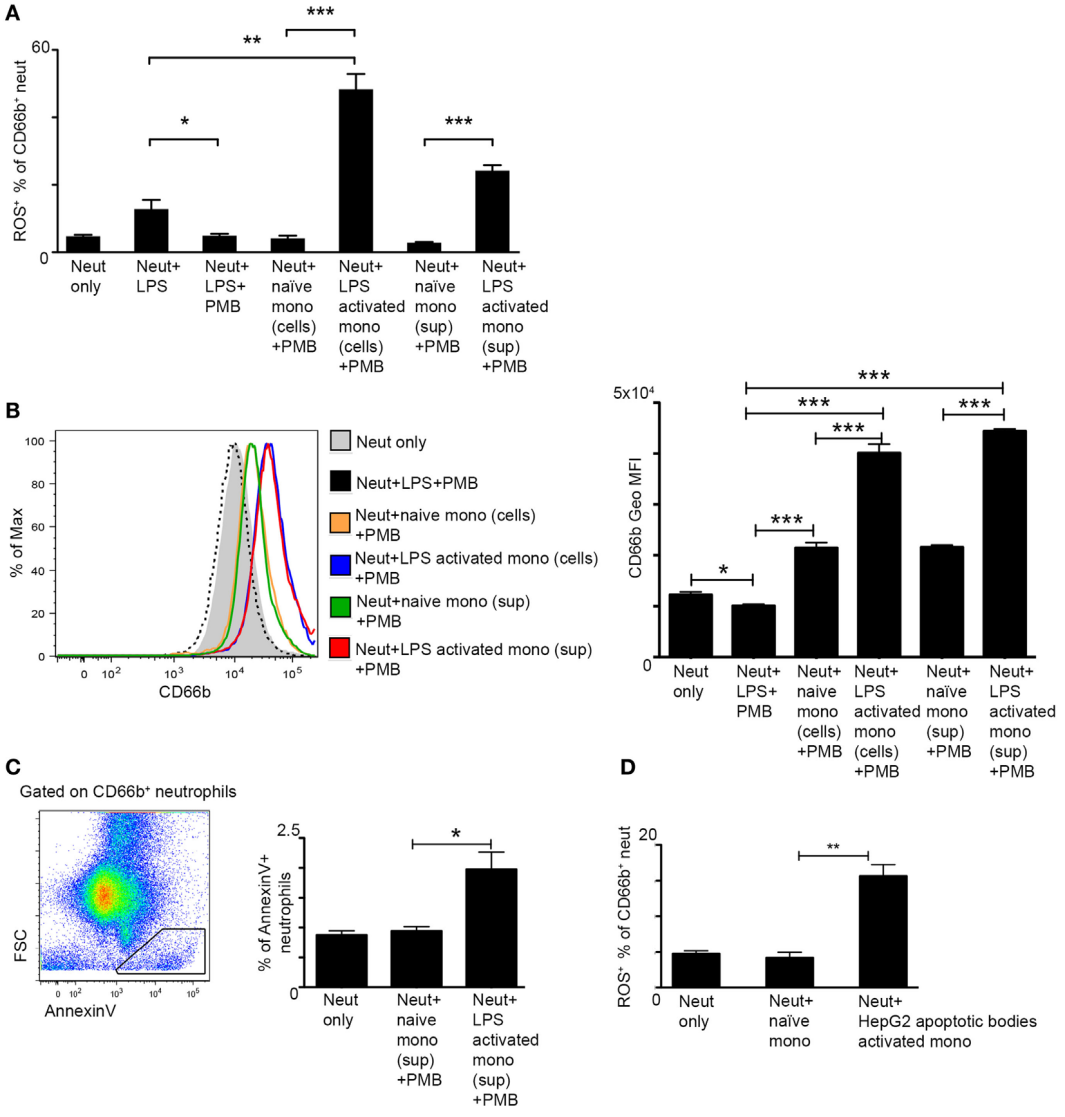
**Activated monocytes directly induce reactive oxygen species (ROS) production by neutrophils**. **(A)** Graphical summary of flow cytometry analyses showing the fraction of ROS^+^ neutrophils out of CD66b^+^ neutrophils following 2 h culture with lipopolysaccharide (LPS)-activated human CD14^+^ monocyte cells or with their cell-free supernatants; Polymyxin B (PMB) was added to all cultures in order to avoid direct activation of neutrophils by LPS remnants. **(B)** Left, representative histogram plot showing the expression level of the neutrophil activation marker CD66b following different treatments. Right, Summarizing graph showing the geometric mean fluorescence intensity (Geo MFI) of CD66b protein following different treatments. **(C)** Left, representative flow cytometry image showing the identification of AnnexinV^+^FSC^lo^ apoptotic cells among CD66b^+^ neutrophils. Right, Summarizing graph showing the fraction (%) of these cells among total CD66b^+^ neutrophils. **(D)** The fraction of ROS^+^ neutrophils out of CD66b^+^ neutrophils following 2 h culture with human CD14^+^ monocyte cells pre-activated with HepG2 apoptotic bodies. Data were analyzed by unpaired, two-tailed *t*-test and are presented as mean ± SEM with significance: *p < 0.05, **p < 0.01, ***p < 0.001 (*n* ≥ 3 human donors/group for each monocyte group, the same pool of neutrophils was used).

In order to examine whether monocytes directly induce ROS production in neutrophils, we performed coculture assays of human CD14^+^ monocytes, the equivalent of the murine Ly6C^hi^ monocytes, and CD66b^+^ neutrophils isolated from the blood of human healthy donors. We resorted to the human setup due to the better survival of these cells following isolation. Monocytes were incubated with medium only or activated with LPS for 2 h, carefully washed to remove LPS, and then cocultured with neutrophils for 2 h. Neutrophil activation was assessed by their production of ROS. PMB was added to the culture in order to exclude any direct activation of neutrophils by LPS remnants. Indeed, PMB was efficient in preventing neutrophil activation even following direct exposure to LPS (Figure 4A). Notably, LPS-stimulated monocytes induced greater ROS production by neutrophils in comparison to naïve monocytes or direct LPS stimulation (Figure 4A). A similar effect was observed in response to 2 h exposure of neutrophils to cell-free supernatants (+ PMB) of activated versus naïve monocytes (Figure 4A). Moreover, neutrophils exposed to LPS-activated monocyte cells or their cell-free supernatants exhibited an activated phenotype as manifested by a significant increase in the protein expression of the neutrophil activation marker CD66b (Figure 4B). Interestingly, AnnexinV^+^ apoptotic cells were more prevalent in neutrophil cultures exposed to supernatants of LPS activated CD14^+^ monocytes in comparison to supernatants of naïve monocytes (Figure 4C). To mimic possible physiological cues encountered by liver infiltrating monocytes during AILI, human CD14^+^ monocytes were cultured for 2 h with hepatocyte apoptotic bodies generated from the human hepatocyte cell line HepG2. Also under these settings, stimulated monocytes induced significant increase in ROS production by the cocultured neutrophils (Figure 4D). Collectively, these results demonstrate the potent ability of monocyte-derived secreted factors to directly activate ROS production by neutrophils.

### Gene Expression Profiling of Neutrophils from Monocyte-Deficient Livers Indicates Altered Innate Immune Functions

**Figure 5:**
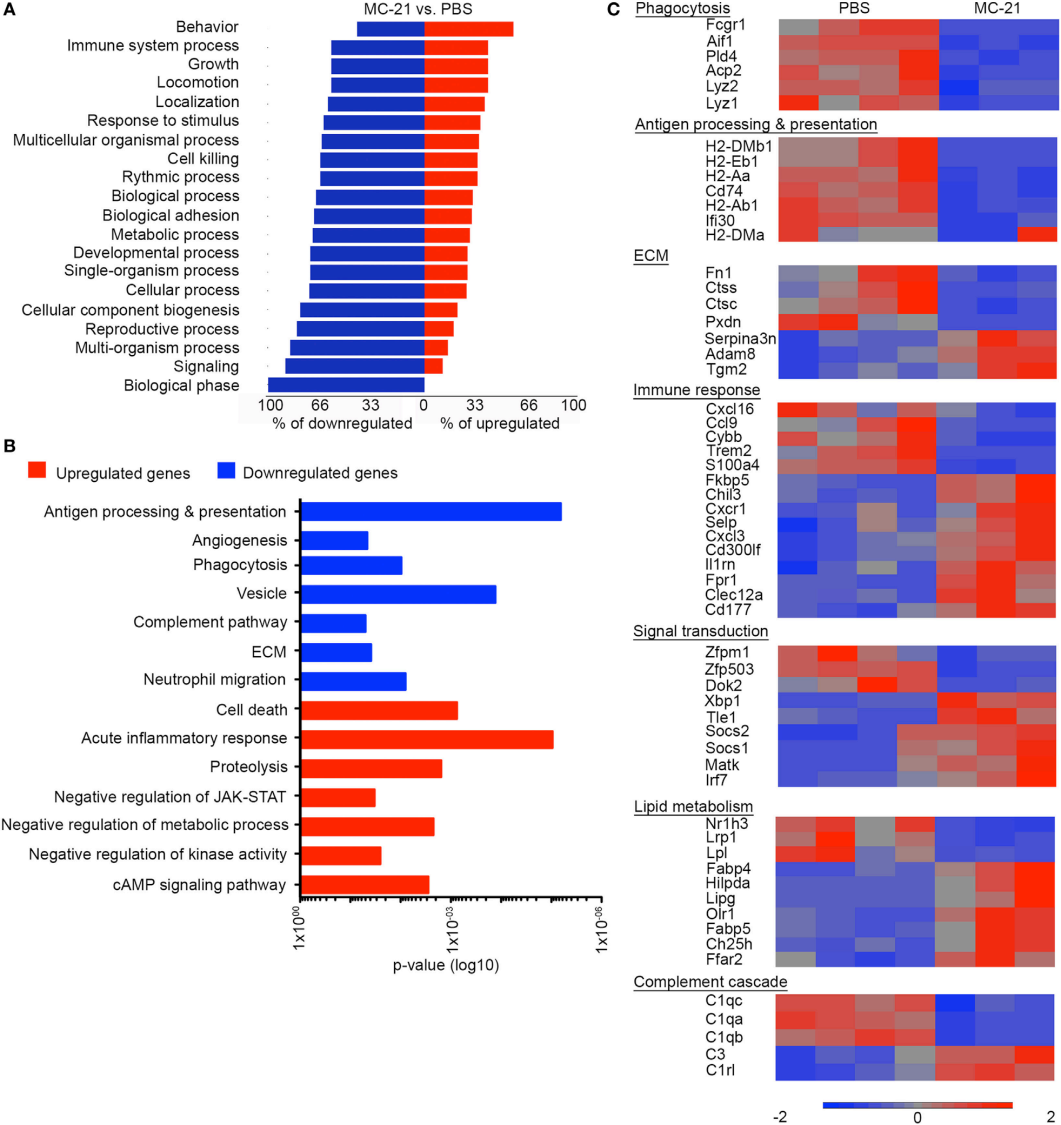
**Altered neutrophil-mediated innate immune functions and cell survival in the absence of Ly6C^hi^ monocytes**. **(A)** Forest plot analysis of 449 differentially expressed genes (*p* < 0.05; *t*-test, with at least twofold change) presenting for each biological process the percentage of downregulated genes (in blue) and upregulated genes (in red). **(B)** Graphic representation of the significance (*p*-value) for the enrichment of selected GO categories out of DAVID analyses performed for the downregulated (in blue) and upregulated genes (in red) comparing between “MC-21” versus “phosphate-buffered saline (PBS)” neutrophils. Analysis was performed on the differentially expressed genes (*p* < 0.05; *t*-test, ≥2-fold change). **(C)** RNA-seq heat map analysis displaying the differential expression (z-scored) of genes between “MC-21” and “PBS” neutrophils, which are associated with phagocytosis, antigen processing and presentation, extracellular matrix (ECM) remodeling, immune response, signal transduction, lipid metabolism, and complement cascade. Color legend is presented at the bottom of the figure. Analysis was performed on the differentially expressed genes (*p* < 0.05; *t*-test, ≥2-fold change).

We next studied the effect of monocyte-absence on neutrophil function. RNA-seq-based gene expression profiling was performed on neutrophils sorted from livers of MC-21- and PBS-treated animals, at 24 h following AILI. Initial analysis revealed 449 genes that were significantly different (*p* < 0.05, *t*-test) with at least twofold change. Forest plot analysis of the differentially expressed genes uncovered a higher percentage of down-regulated genes for each functional group (Figure 5A), suggesting an overall decreased activity of “MC-21 neutrophils.” Utilizing the DAVID bioinformatics database, we revealed among the downregulated genes a functional enrichment for biological processes, such as antigen processing and presentation, angiogenesis, phagocytosis, complement pathway, extracellular matrix (ECM) remodeling, and neutrophil migration. In contrast, the list of upregulated genes displayed enrichment for genes associated with regulation of cell death, acute inflammatory response, proteolysis, negative regulation of JAK-STAT cascade, negative regulation of metabolic processes, negative regulation of kinase activity, and cAMP signaling pathway (Figure 5B).

In depth gene expression comparison further supported the idea that monocytes positively regulate innate immune activity of colocalized neutrophils (Figure 5C). In alignment with the reduction in ROS-producing neutrophils in the absence of monocytes (Figure 3), the gene encoding for NADPH Oxidase 2 (*Cybb*) was significantly reduced in “MC-21 neutrophils” in comparison with “PBS neutrophils” (Figure 5C). Neutrophils also serve as rapid and potent phagocytes during tissue regeneration; however, “MC-21 neutrophils” displayed decreased expression of phagocytosis genes including the lysozymes *Lyz1* and *Lyz2*, the actin binding protein allograft inflammatory factor 1 (*Aif1*), the phospholipase D4 (*Pld4*), the lysosomal acid phosphatase 2 (*Acp2*), and the Fc gamma receptor-1 CD64 (*Fcgr1*). On a different note, up-regulation of genes associated with antigen processing and presentation are often identified in activated neutrophils under certain inflammatory scenes (51–53). In support of their lower activation, “MC-21 neutrophils” exhibited clear reduction in the expression of several MHC class II molecules, including *H2-Aa, H2-Ab1, H2-DMa*, and *H2-DMb1*, the CD74 invariant chain of MHCII (*Cd74*) as well as the lysosomal thiol reductase *Ifi30*. Indeed, flow cytometry analysis confirmed a reduction in the fraction of MHCII^+^ neutrophils following Ly6C^hi^ monocyte ablation (Figure S2 in Supplementary Material). With respect to tissue ECM remodeling features, “MC-21 neutrophils” had lower expression of cathepsins C and S (*Ctsc* and *Ctss*), fibronectin (*Fn1*), and heme-containing peroxidase (*Pxdn*), though they had higher expression of the ECM covalent cross-linker transglutaminase 2 (*Tgm2*), and a disintegrin and metalloproteinase 8 (*Adam8*).

Notably, transcriptomic profiling of “MC-21 neutrophils” also indicated an overall reduced inflammatory activity (Figure 5C). Accordingly, there was elevated expression of anti-inflammatory transcriptional regulators, including the suppressor of cytokine signaling 1 (*Socs1*) and 2 (*Socs2*), which negatively regulate cytokine-induced signaling through the JAK/STAT3 pathway, the megakaryocyte-associated tyrosine kinase (*Matk*), which negatively regulates Src family kinases, and the transducing-like enhancer of split 1 (*Tle1*), which is a suppressor of NFkB transcriptional activity (54). Furthermore, nuclear factor kappa B subunit 2 (*Nfkb2*) was significantly reduced in “MC-21 neutrophils” (1.3-fold-change, *p* = 0.0001) (data not shown). They have also displayed higher levels of myeloid inhibitory receptors that carry tyrosine-based inhibitory motifs (ITIMs), such as CD300 molecule-like family member F (*Cd300lf*) and C-type lectin domain family 12 member A (*Clec12A*). In addition, they had increased expression of the inhibitor interleukin 1 receptor antagonist (*Il1rn*) and of the FK506 Binding Protein 5 (*Fkbp5*); the latter was shown to play a key role in the immune suppressive activity of tumor associated suppressor granulocytes (55).

Intriguingly, “MC-21 neutrophils” exhibited altered expression of genes related to lipid metabolism. Specifically, there was upregulation in the gene expression of the oxidized low-density lipoprotein receptor 1 (*Olr1*), which drives the internalization of oxidized-LDL. There was also increased expression of the free fatty acid transporters, fatty acid binding protein 4 (*Fabp4*), and 5 (*Fabp5*). In contrast, there was a reduction in the expression of genes involved with triglyceride uptake, including the low-density lipoprotein receptor-related protein 1 (*Lrp1*), which drives the uptake of triglycerides rich very low-density lipoproteins (VLDLs), and lipoprotein lipase (*Lpl*), which catalyzes the hydrolysis of triglycerides. Concomitantly with the reduced triglycerides uptake, there was an increased expression of hypoxia-inducible lipid droplet-associated (*Hilpda*), which induces intracellular triglyceride storage through the inhibition of VLDL secretion. “MC-21 neutrophils” also displayed an altered expression of lipid and cholesterol metabolism regulators, including increased expression of the enzymes lipase G (*Lipg*) and cholesterol 25-hydroxylase (*Ch25h*), and reduced expression of the nuclear receptor Liver X receptor alpha (LXRα, *Nr1h3)* (Figure 5C). Collectively, these results suggest that Ly6C^hi^ monocytes induce in neutrophils transcriptional changes that are overall associated with increased inflammatory phenotype and activity.

### Ly6C^hi^ Monocytes and their MoMF Descendants Mediate Neutrophil Apoptosis and Clearance, Respectively

**Figure 6:**
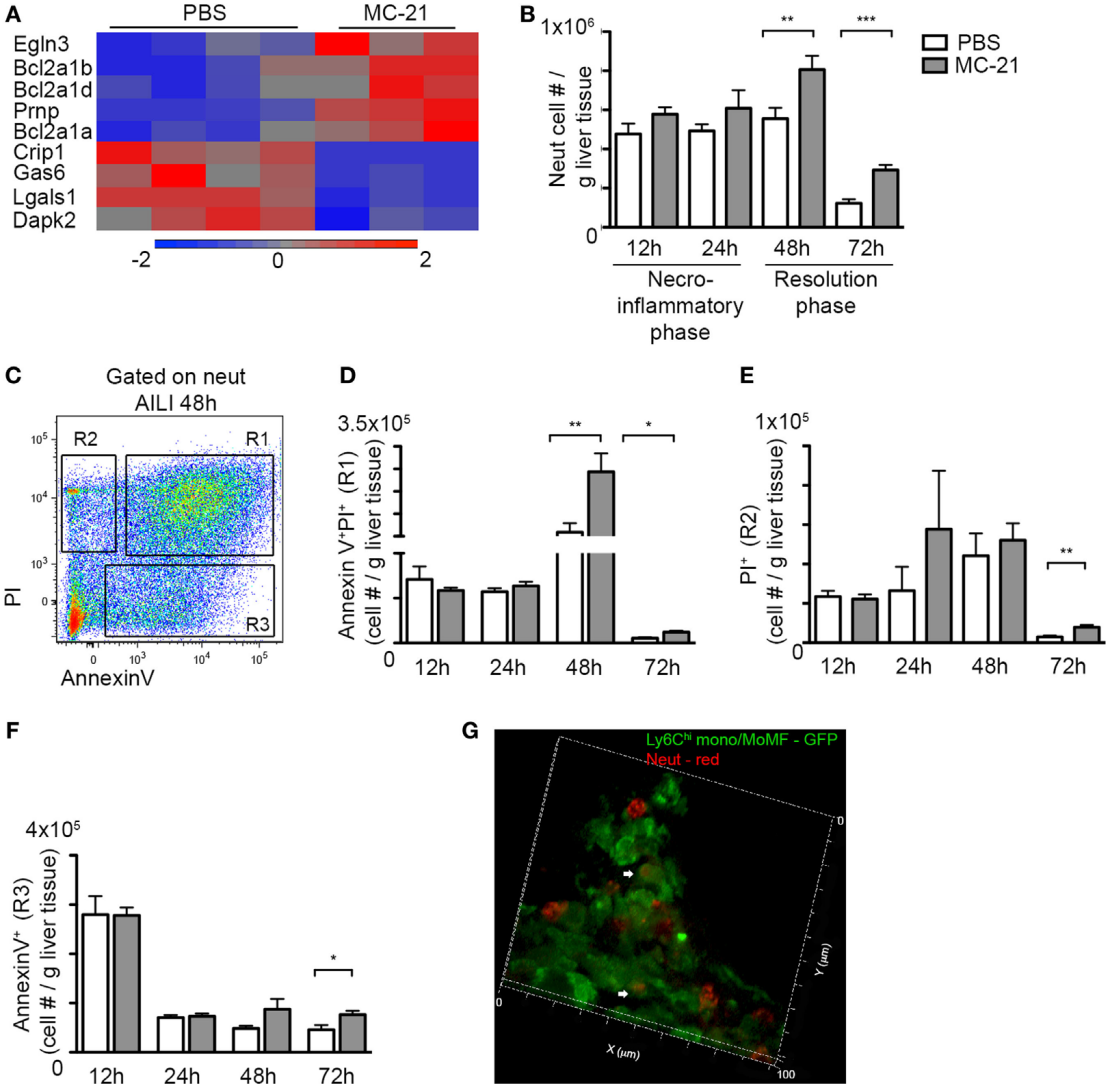
**Delayed clearance of apoptotic neutrophils in the absence of Ly6C^hi^ monocytes and monocyte-derived macrophages (MoMF)**. **(A)** RNA-seq heat map analysis displaying the differential expression (*z*-scored) of genes between “MC-21” and “phosphate-buffered saline (PBS)” neutrophils, which are associated with cell survival regulation. **(B)** Graphic representation of neutrophil numbers normalized per liver tissue mass (in gram) in the injured liver in presence (PBS) and absence of Ly6C^hi^ monocytes and their MoMF descendants (MC-21), as analyzed by flow cytometry. **(C)** Flow cytometry image showing the discrimination between early apoptotic (AnnexinV^+^), late apoptotic (AnnexinV^+^PI^+^), and necrotic (PI^+^) neutrophils at 48 h following acetaminophen-induced liver injury (AILI). **(D–F)** Graphical summaries showing cell counts normalized per liver tissue mass (in gram) of **(D)** late apoptotic neutrophils, **(E)** necrotic neutrophils, and **(F)** early apoptotic neutrophils. **(G)** 3D-reconstructed confocal image generated from 20 µm *Z*-stacks of livers sections extracted at AILI-48 h from *Cx3cr1^gfp/+^* mice. Ly6C^hi^ monocyte-derived cells are in GFP and neutrophils are in red. Magnification ×63. Heat map analysis in A was performed on the differentially expressed genes (*p* < 0.05; t-test, ≥2-fold change). Data in **(B–F)** were analyzed by unpaired, two-tailed *t*-test, comparing at each time point livers from PBS (white) versus MC-21 (gray)-treated mice and are presented as mean ± SEM with significance: *p < 0.05, **p < 0.01, and ***p < 0.001 (*n* ≥ 5 mice/group for each time point).

Once neutrophils exerted their function, they launch apoptotic pathways that ensure clearance of ROS-producing neutrophils from injured tissue to avoid excessive inflammation and oxidative damage (29, 56). RNA-seq analysis at the necroinflammatory phase revealed that neutrophils upregulate anti-apoptotic genes and downregulate proapoptotic genes in the absence of Ly6C^hi^ monocytes (Figure 6A). Striking was the upregulation of Bcl-2A1 genes (*Bcl2a1a, Bcl2a1b*, and *Bcl2a1d*) that promote neutrophil survival (57–59). There was also downregulation in the expression of proapoptotic mediators, including the serine/threonine kinase death-associated protein kinase 2 (*Dapk2*) and galectin-1 (*Lgals1*) (Figure 6A). Together with the findings that activated CD14^+^ monocytes induce ROS production by neutrophils and the fraction of AnnexinV^+^ apoptotic neutrophils (Figure 4), these gene expression alterations suggest that Ly6C^hi^ monocytes facilitate neutrophil apoptosis.

Therefore, we next sought to investigate how Ly6C^hi^ monocyte and MoMF ablation influence neutrophil survival. Interestingly, monocyte ablation had no impact on neutrophil numbers in the liver at 12 and 24 h following AILI (Figure 6B). In contrast, there was a significant increase in neutrophil numbers in the resolution phase at 48 and 72 h following AILI (Figure 6B), which coincided with the conversion of Ly6C^hi^ monocytes into MoMF (Figure 1E). It also coincided with the accumulation of ROS^−^ neutrophils, which were smaller and less granular in comparison with the ROS^+^ (Figures 3F,G). We next stained hepatic non-parenchymal cells from injured livers of MC-21-treated mice and controls at different time points after AILI for cellular markers of apoptosis and necrosis (Figure 6C). Selective ablation of Ly6C^hi^ monocytes had no effect on the frequency of apoptotic or necrotic neutrophils during the necroinflammatory phase (Figures 6D–F). Yet, there was a significant accumulation of late apoptotic AnnexinV^+^PI^+^ neutrophils at both 48 and 72 h post-AILI (Figure 6D) and an increase of PI^+^ necrotic and AnnexinV^+^ apoptotic neutrophils at 72 h post-AILI (Figures 6E,F). High-resolution confocal imaging further exposed the internalization of Ly6G^+^ neutrophils by Ly6C^hi^ monocyte-derived cells at AILI-48 h (Figure 6G, Movie S2 in Supplementary Material), most probably MoMF that dominate this early phase of resolution (Figure 1E). Therefore, these results suggest continued involvement of Ly6C^hi^ monocytes and their MoMF descendants in the regulation of neutrophil apoptosis and clearance.

### MoMF Express a Unique Set of Apoptotic Cell Bridge Molecules and Receptors

**Figure 7:**
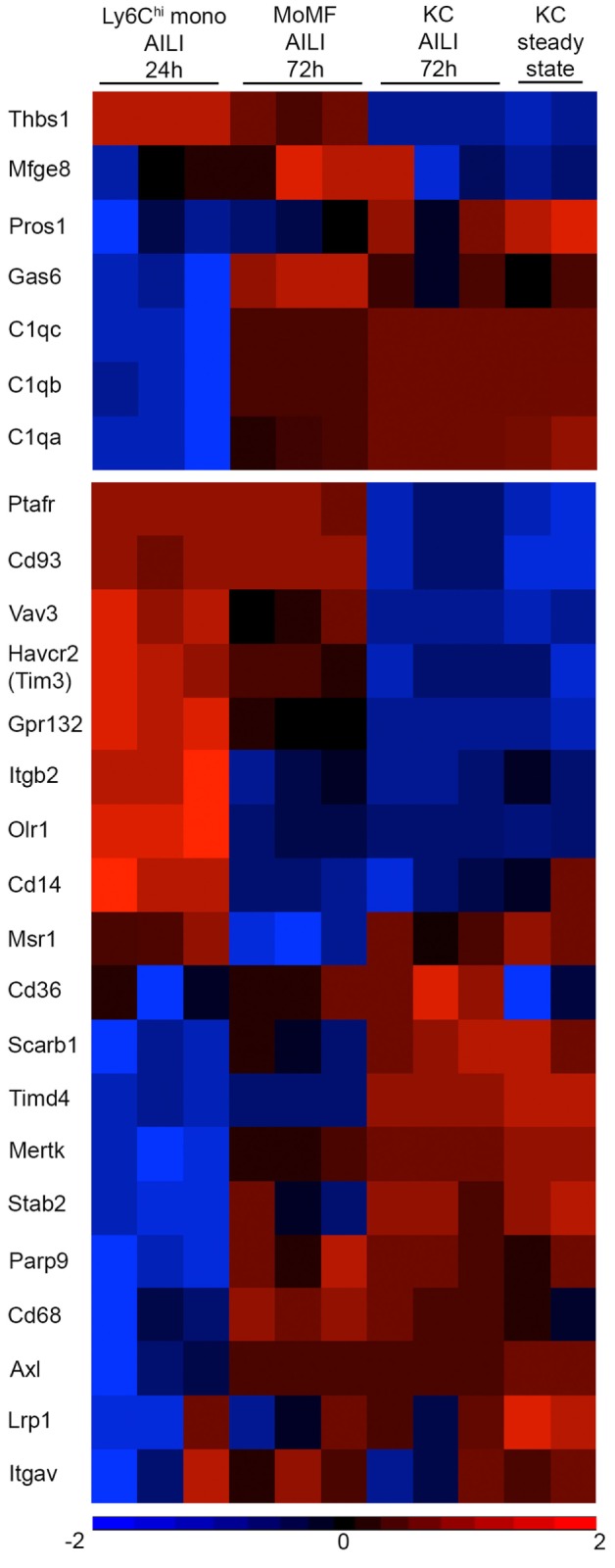
**Variable expression of apoptotic cell bridging molecules and receptors between Ly6C^hi^ monocytes, monocyte-derived macrophages (MoMF), and Kupffer cell (KC)**. Heat map analysis generated using Partek Genomics Suite version 6.6 (Partek, St. Louis, MO, USA). The heat maps show the fold-change gene-expression differences of bridge molecules (upper panel) and macrophage receptors (lower panel) involved with apoptotic cell clearance. The heat map presents comparison between Ly6C^hi^ monocytes [acetaminophen-induced liver injury (AILI) 24 h], MoMF (AILI 72 h), and KC (steady state and AILI 72 h). Color legend is presented at the bottom of the figure. Statistical significance for specific genes is mentioned as *p*-value in the text and was analyzed by ANOVA followed by Bonferroni’s multiple comparison test. These results were analyzed out of a published database (22).

Recognition of apoptotic cells is performed by an increasing number of bridge molecules and macrophage receptors (29). We previously performed a comprehensive microarray-based molecular profiling of Ly6C^hi^ infiltrating liver monocytes sorted from APAP 24 h livers, MoMF sorted from APAP 72 h livers, and KC sorted from steady state and APAP 72 h livers (22). Mining out of this database revealed their variable expression of bridge molecules and receptors involved with the engulfment of apoptotic cells (Figure 7). Specifically, upon their differentiation into MoMF, Ly6C^hi^ monocytes significantly upregulated the expression of the TAM receptor protein tyrosine kinases Mertk and Axl (*p* = 0.0001 and 0.01, respectively) and their bridging molecule *Gas6* (*p* = 0.0001), as well as the gene expression of C1qa, b, and c subunits of the complement complex C1q (*p* = 0.0001, 0.0001, and 7.90E−05, respectively). Interestingly, C1q complement complex genes were also reduced in the MC-21 neutrophils (Figure 5C). MoMF were also significantly higher for the C1q-receptor CD93 compared to recovering KC (*p* = 0.0001). Moreover, MoMF expressed the bridging molecule milk fat globule-EGF factor 8 gene (*Mfge8*) (Figure 7) and CX_3_CR1 (Figure 1C). Of note, CX_3_CL1 is suggested to induce the clearance of apoptotic cells through the induction of MFG-E8 (60).

Notably, clearance of late apoptotic neutrophils was eventually accomplished between 48 and 72 h in spite of the absence of MoMF (Figure 6D). This is at the time, when resident KC starts recovering (Figure 1D). Indeed, both steady state and recovering KC expressed a large variety of apoptotic cell bridge molecules and engulfment receptors, but the recovered KC were significantly higher for the oxidized PS scavenger receptor CD36 (*p* = 0.005) (Figure 7), suggesting its possible involvement in apoptotic neutrophil clearance. Nevertheless, our results show that neutrophils accumulate mostly in the centrilobular areas of necrosis (Figures 1A,B), while KC mainly localize to liver sinusoids. Finally, the expression of G2A receptor (*Gpr132*), which drives the clearance of dying neutrophils through its binding to lyso-phosphatidylserine (lyso-PS) (61), was significantly higher in Ly6C^hi^ monocytes than in MoMF (*p* = 5.7E−05) and recovering KC (*p* = 1.4E−06). Ly6C^hi^ monocytes were also significantly higher for the bridge molecule thrombospondin-1 (*Thbs1*) compared to MoMF (*p* = 0.009) and KC (*p* = 6.1E−06) (Figure 7). While the ablation of Ly6C^hi^ monocytes had no effect on the accumulation of dying or necrotic neutrophils during the necroinflammatory phase of AILI (Figure 6), they still may be involved in neutrophil clearance at 24–48 h. Noteworthy, G2A and THBS1 gene expression levels remained significantly higher on MoMF versus recovering KC (*p* = 0.0003 and *p* = 6E−05, respectively), implying on their possible involvement in MoMF-governed neutrophil clearance during early resolution phase.

## Discussion

The concerted action of professional phagocytes, including tissue-resident macrophages, recruited monocytes, and neutrophils, is fundamental for the effective elimination of noxious agents and the restoration of tissue homeostasis after injury or infection (28). Our data uncover a new immunoregulatory role for Ly6C^hi^ monocytes and their MoMF progenies by their regulation of neutrophil activity and clearance during AILI. Specifically, we demonstrate that liver-infiltrating Ly6C^hi^ monocytes activate ROS production in neutrophils in a direct manner. Further transcriptomic profiling implies that Ly6C^hi^ monocytes positively regulate neutrophil-mediated phagocytosis and inflammation. It also suggests a role for monocytes in the induction of apoptotic pathways in colocalized neutrophils. At the resolution phase, MoMF play a major role in the clearance of apoptotic neutrophils through their expression of a unique set of apoptotic cell recognition bridge molecules and receptors.

The division of labor between tissue-resident and monocyte-derived macrophage subsets in the resolution from injuries is under intense investigation (1). While it is well established that tissue-resident macrophages are critically involved in the initial recognition of tissue damage and the subsequent recruitment of inflammatory neutrophils and monocytes (28), emerging evidences in gut (49, 62) and liver (22) inflammation suggest that resident macrophages are also robustly imprinted to resist stimuli associated with acute inflammation. In contrast, monocytes display extreme functional plasticity and their immediate availability in the circulation makes them well-suited for a rapid recruitment and performance of acute effector functions required for promoting the initiation, propagation, and resolution of tissue inflammation (2). Indeed, monocytes were shown to play a critical role in the inflammatory and recovery phases of different tissue-specific injuries (1, 17–20). In liver fibrosis, Ly6C^hi^ monocytes produce proinflammatory mediators that promote hepatic stellate cell activation and fibrosis (7), but subsequently give rise to prorestorative Ly6C^lo^ macrophages (5, 8). Similarly, we have demonstrated in an acute model of AILI that recruited Ly6C^hi^ monocytes differentiate into distinct short-lived prorestorative MoMF that outnumber the resident KC population at the early recovery phase. Transcriptomic profiling revealed that Ly6C^hi^ monocytes activate upon their differentiation into MoMF molecular pathways that are associated with regenerative functions, including among others tissue scavenging, angiogenesis, and ECM remodeling (22).

Here, we provide a more detailed comprehension of the interplay between liver infiltrating monocytes and neutrophils, which spatially and temporally overlap in the centrilobular necrotic areas following AILI. During the initial inflammatory phase, monocytes and neutrophils become the dominant phagocyte subsets in the injured liver. Selective ablation of Ly6C^hi^ monocytes has no effect on neutrophil generation or recruitment, as evident by their similar representation in the circulation and in the liver tissue during the first 24 h. However, in the absence of Ly6C^hi^ monocytes, we observe a significant reduction in neutrophil activation during the inflammatory phase, as manifested by reduced ROS production, a hallmark of neutrophil activation (26–29). We also show reduced expression of NADPH oxidase 2 in the “MC-21 neutrophils,” a key mediator of neutrophil-driven oxidative burst (50). Coculture assays of stimulated CD14^+^ human monocytes with naïve neutrophils further reveal that monocyte-mediated neutrophil activation is imprinted by a cell-intrinsic and contact-independent mechanism. Monocyte stimulation with LPS or with apoptotic bodies of human hepatocytes, the latter as an example of environmental cues that monocytes encounter at the necrotic areas of the injured liver, can both induce significant activation of cocultured neutrophils as manifested by increased ROS production and augmented expression of the neutrophil activation marker CD66b (63). Notably, cell-free supernatants could also activate ROS production in the naïve neutrophils suggesting that monocyte-secreted mediators are likely to regulate such interaction. Of note, our results are in dispute with a previous study performed in a murine model of intestinal parasite infection (64). In that study, the authors elegantly demonstrated that Ly6C^hi^ monocytes shut down ROS production in neutrophils that are recruited to the infected tissue (64). Given the plasticity of monocytes, there might be distinct environmental cues that affect monocyte ability to control neutrophil activation. The nature of the potentially involved monocyte-derived factors remains to be defined, but may include cytokines, and also lipid mediators (65). With respect to the latter, we have previously shown that Ly6C^hi^ monocytes uniquely express the Ptgs2 gene, which encodes for cyclooxygenase-2 (COX2), and the microsomal PGE synthase-1 gene (*Ptges*), both of which constitute key enzymes in PGE2 synthesis (22).

RNA-seq profiling comparing between neutrophils from normal versus monocyte-deficient livers at the necroinflammatory phase further reinforces our claim that Ly6C^hi^ monocyte-derived signals activate innate immune functions in neutrophils. During tissue injury, recruited neutrophils play key role in the removal of damaged cells and cellular debris and prepare the tissue for regeneration (26–29). We show the inability of neutrophils to upregulate key genes involved with phagocytosis in the absence of Ly6C^hi^ monocytes. Induction of genes related to antigen presentation has also been noted in neutrophils under different forms of activation (51–53). Our results imply that monocyte-derived substances induce the expression of MHCII genes in neutrophils. While these results do not necessarily attribute antigen presentation capabilities to neutrophils, they do provide another marker of neutrophil activation that is reduced in the absence of monocytes. Moreover, neutrophils acquire an anti-inflammatory phenotype in the absence of monocytes. This is manifested by the upregulation of various anti-inflammatory transcription factors and myeloid inhibitory receptors in response to Ly6C^hi^ monocyte ablation. Intriguingly, we show altered expression of genes involved in the uptake of modified lipoproteins, breakdown and storage of triglycerides as well as transport and metabolism of cholesterol. While these changes are well-characterized in macrophages, especially in atherosclerosis, there are only sporadic evidences for similar gene expression alterations in activated neutrophils (51), and their mechanistic involvement in tissue injury and resolution is unclear.

Neutrophil activation has to be tightly controlled to avoid excessive tissue damage. In terms of resolution, apoptosis of neutrophils prevents further neutrophil recruitment and terminates their production of deleterious substances. We demonstrate a profound accumulation of AnnexinV^+^PI^+^ late apoptotic neutrophils specifically during the early resolution phase. Previous studies have indicated that ROS and oxidative stress can lead to neutrophil apoptosis through disruption of the mitochondria transmembrane potential [reviewed in Ref. (66)], suggesting that Ly6C^hi^ monocyte-induced ROS production by neutrophils may facilitate their apoptosis. Indeed, we show that in the absence of monocytes, neutrophils exhibit reduction in proapoptotic genes and elevation in cell survival molecules such as BCL21A. Moreover, supernatants from LPS-activated monocytes directly increase the fraction of AnnexinV^+^ apoptotic neutrophils. The phagocytic removal of apoptotic neutrophils is an additional mechanism to clear effete neutrophils and ultimately facilitate the resolution of inflammation. We show here that concomitantly with the need for clearance of apoptotic neutrophils at the early resolution phase, Ly6C^hi^ monocytes differentiate into MoMF, which become the dominant macrophage subset at this stage. Our gene-expression results further uncover that Ly6C^hi^ monocytes upregulate the expression of various apoptotic cell recognition bridge molecules and receptors upon their differentiation into MoMF, which may qualify the latter for the clearance of apoptotic neutrophils. Notably, even in the absence of MoMF, there is still clearance of late apoptotic neutrophil levels between 48 and 72 h following AILI, suggesting that other cells are also likely to take part in the neutrophil removal process, such as KC, which start repopulating at that time. We show that the recovering KC population also expresses a wide variety of apoptotic cell bridge molecules and receptors, some shared with MoMF and others are unique. Indeed, the combined absence of KC and MoMF results in a marked delay in liver repair, greater than each one alone (24).

We have previously reported that inducible and selective monocyte ablation results in impaired liver regeneration, highlighting MoMF as pivotal players in the resolution from liver injury (22). We showed that already at the necroinflammatory phase, Ly6C^hi^ monocyte ablation aggravates hepatic damage. In contrast, here we demonstrate that Ly6C^hi^ monocyte ablation at both 12 and 24 h following acute AILI has no significant impact on hepatic damage. This is further supported by a similar elevation in the serum levels of ALT and AST liver enzymes during the first 24 h of AILI between PBS and MC-21-treated mice. The discrepancy in these results may be related to the different routes of APAP administration implemented in these studies. As Ly6C^hi^ monocytes express both proinflammatory and restorative genes (22), their ablation may concomitantly interfere with inflammation-induced damage and resolution. Nevertheless, these results suggest that Ly6C^hi^ monocyte-mediated regulation of neutrophil activity, and specifically promotion of ROS production, does not significantly contribute to hepatocyte damage. This is in alignment with a previous study reporting that neutrophils do not contribute to the initiation or progression of hepatic damage during acute AILI (35). Importantly, we corroborate our previous findings (22) demonstrating that ablation of Ly6C^hi^ monocytes and their MoMF descendants impair liver resolution at 48 h following AILI. We also show that their ablation leads to increased accumulation of late apoptotic neutrophils. Therefore, it may be that this delay in neutrophil clearance perpetuates the inflammatory response and interferes with hepatic resolution. Of note, MoMF express large repertoire of prorestorative factors promoting hepatocyte growth, ECM remodeling, and angiogenesis (22). Thus, it is has difficult to determine to what extent the altered neutrophil activity and clearance contribute to the increased liver damage following monocyte and MoMF ablation.

Collectively, our results suggest a sequence of interactions following AILI between Ly6C^hi^ monocytes, MoMF, and neutrophils. We show that Ly6C^hi^ monocytes promote neutrophil activation at the injury site during the initial necroinflammatory phase, an important step for the removal of damaged tissue. Subsequently, ROS production in neutrophils may facilitate their apoptosis and their subsequent clearance by MoMF.

## Author Contributions

The corresponding author (CV) confirms that all authors agree to be accountable for the content of the work. NG and CV designed, performed, and analyzed all experiments and wrote the manuscript. MV, OM, GC, and DR substantially assisted NG with some of the major *in vivo* experiments. LCM performed the RNA library generation for the RNA-seq analysis and MPC and ED analyzed the gene expression RNA-Seq data. EB performed the histopathological assessments. EZ contributed to the design of the original idea in this manuscript. SJ mentored NG together with CV, contributed to the experimental design and analysis and critically reviewed the manuscript.

## Conflict of Interest Statement

The research was conducted in the absence of any commercial or financial relationships that could be construed as a potential conflict of interest.

## Footnotes

^1^http://www.partek.com/pgs.

^2^http://www.ncbi.nlm.nih.gov/geo.

## Supplementary Material

Figure S1 **Ablation of Ly6C^hi^ monocytes and their monocyte-derived macrophage descendants does not affect liver damage during the necro-inflammatory phase of acetaminophen-induced liver injury (AILI), but attenuates liver resolution**. **(A)** Hematoxylin and eosin (H&E) of liver sections at 12, 24, and 48 h following AILI. Original magnification ×10. Bars, 200 µm. Note the extended necrotic area at 48 h. **(B)** Histopathological score at 12, 24, and 48 h following AILI of mice injected with PBS (Red circle) or MC-21 (Blue open square). **(C)** ALT and **(D)** AST levels in the blood of MC-21-injected mice compared to control mice at steady state (SS) and 24, 48, 72, and 120 h after AILI. Data were analyzed by unpaired, two-tailed *t*-test and presented as mean ± SEM with significance: **p* < 0.05. Experiments were repeated at least three times, *n* ≥ 3 mice per group.

Figure S2 **Ablation of Ly6C^hi^ monocytes reduces MHCII protein expression in neutrophils**. Flow cytometry analysis showing MHCII expression by Ly6G^+^ neutrophils extracted from normal (PBS) or Ly6C^hi^ monocyte-ablated livers (MC-21), at 24 h following acetaminophen-induced liver injury. Below, graphical summary of the fraction of MHCII^+^ neutrophils out of total neutrophils.

Movie S1 **Colocalization of liver infiltrating Ly6C^hi^ monocyte-derived cells and neutrophils**. A representing movie of 3D reconstructed confocal images extracted from 20 µm *Z*-stacks at acetaminophen-induced liver injury 48 h livers of Cx3cr1^gfp/+^ mice. Magnification ×63. The movie demonstrates the close proximity of Ly6C^hi^ monocyte-derived cells (GFP) and Ly6G^+^ neutrophils (red).

Movie S2 **Liver infiltrating Ly6C^hi^ monocyte-derived cells uptake neutrophils at the resolution phase**. A representing movie of 3D reconstructed confocal images extracted from 20 µm *Z*-stacks at acetaminophen-induced liver injury-48 h livers of Cx3cr1^gfp/+^ mice. Note that some of the Ly6C^hi^ monocyte-derived cells (GFP) have engulfed neutrophils stained for Ly6G (in red). Magnification ×63.

## Abbreviations

AILI, acetaminophen-induced liver injury; ALT, alanine aminotransferase; APAP, *N*-acetyl-p-aminophenol; AST, aspartate aminotransferase; BM, bone marrow; KCs, Kupffer cells; MoMF, monocyte-derived macrophages; PMB, polymixin B; ROS, reactive oxygen species.
