# Supplementary figures and images for: Ly6Chi Monocytes and Their Macrophage Descendants Regulate Neutrophil Function and Clearance in Acetaminophen-Induced Liver Injury
Source: Front Immunol. 2017 Jun 1;8:626. doi: 10.3389/fimmu.2017.00626 (PMC5451509; doi:10.3389/fimmu.2017.00626)

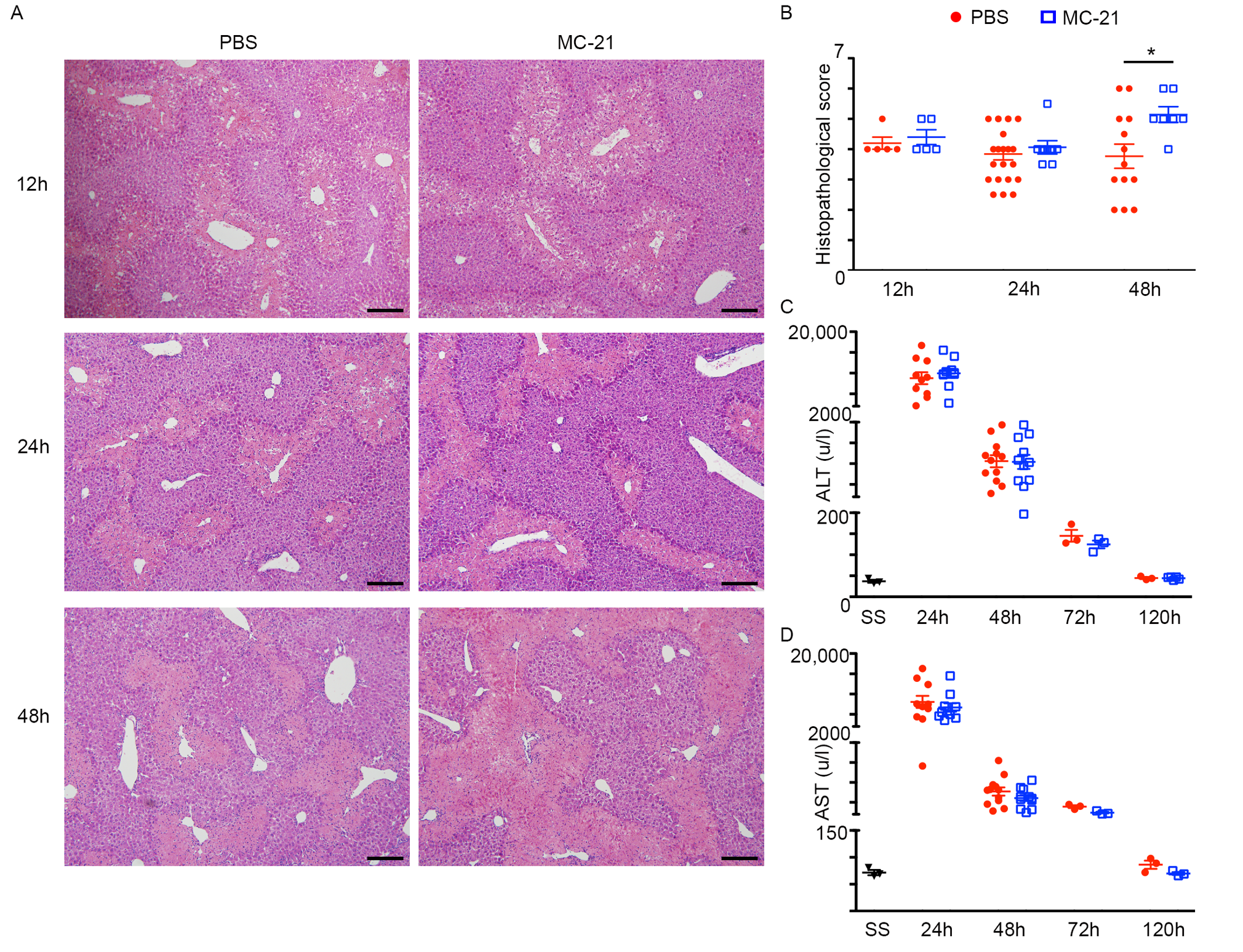

Supplement: Figure S1 — Ablation of Ly6Chi monocytes and their monocyte-derived macrophage descendants does not affect liver damage during the necro-inflammatory phase of acetaminophen-induced liver injury (AILI), but attenuates liver resolution. (A) Hematoxylin and eosin (H&E) of liver sections at 12, 24, and 48 h following AILI. Original magnification ×10. Bars, 200 µm. Note the extended necrotic area at 48 h. (B) Histopathological score at 12, 24, and 48 h following AILI of mice injected with PBS (Red circle) or MC-21 (Blue open square). (C) ALT and (D) AST levels in the blood of MC-21-injected mice compared to control mice at steady state (SS) and 24, 48, 72, and 120 h after AILI. Data were analyzed by unpaired, two-tailed t-test and presented as mean ± SEM with significance: *p < 0.05. Experiments were repeated at least three times, n ≥ 3 mice per group. [file Image_1.TIF]

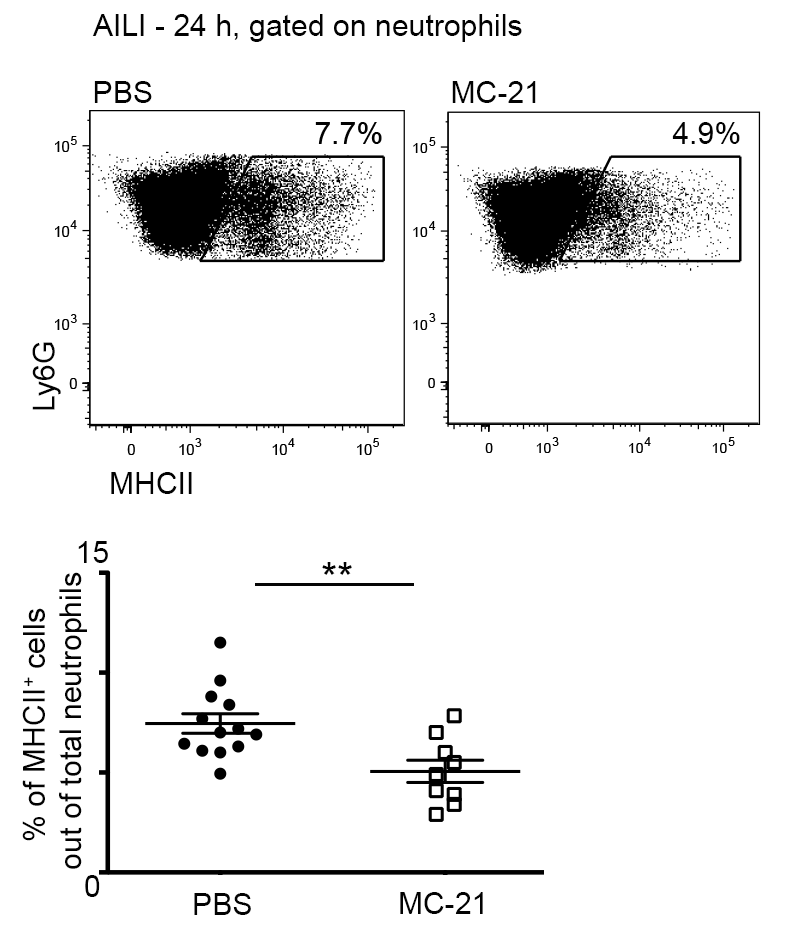

Supplement: Figure S2 — Ablation of Ly6Chi monocytes reduces MHCII protein expression in neutrophils. Flow cytometry analysis showing MHCII expression by Ly6G+ neutrophils extracted from normal (PBS) or Ly6Chi monocyte-ablated livers (MC-21), at 24 h following acetaminophen-induced liver injury. Below, graphical summary of the fraction of MHCII+ neutrophils out of total neutrophils. [file Image_2.TIF]
